# Oral Bioavailability and Lymphatic Transport of Pueraria Flavone-Loaded Self-Emulsifying Drug-Delivery Systems Containing Sodium Taurocholate in Rats

**DOI:** 10.3390/pharmaceutics10030147

**Published:** 2018-09-05

**Authors:** Jin Qiao, Danyang Ji, Shilin Sun, Guangyuan Zhang, Xin Liu, Bingxue Sun, Qingxiang Guan

**Affiliations:** 1 School of Pharmacy, Jilin University, Changchun 13002, China; qiaojin17@mails.jlu.edu.cn (J.Q.); jidy16@jlu.edu.cn (D.J.); zhanggy17@mails.jlu.edu.cn (G.Z.); liux@jlu.edu.cn (X.L.); sunbingxue@jlu.edu.cn (B.S.); 2 Jilin Institute for Drug Control, 657 Zhanjiang Road, Changchun 130031, China; sunsl14@jlu.edu.cn

**Keywords:** SMEDDS, pueraria flavone, in vitro release, relative bioavailability, lymphatic transport

## Abstract

We developed self-microemulsifying drug-delivery systems (SMEDDS), including bile salts, to improve the oral bioavailability of pueraria flavones (PFs). The physical properties of the SMEDDS using Cremophor RH 40, and bile salts as mixed surfactants at weight ratios of 10:0–0:10 were determined. The particle sizes of PFs-SMEDDS_NR_ containing sodium taurocholate (NaTC) and Cremophor RH 40, and PFs-SMEDDS_R_ containing Cremophor RH 40 were measured upon dilution with deionized water and other aqueous media. Dilution volume presented no remarkable effects on particle size, whereas dilution media slightly influenced particle size. PFs-SMEDDS_NR_ and PFs-SMEDDS_R_ provided similar release rates in pH-1.2 hydrochloride solution. However, the release rate of PFs-SMEDDS_NR_ was faster than that of PFs-SMEDDS_R_ in pH-6.8 phosphate buffer containing 20 mM NaTC and 500 U/mL porcine pancreas lipase. The pharmacokinetics and bioavailability were measured in rats. The oral bioavailability of PFs-SMEDDS_NR_ was 2.57- and 2.28-fold that of a suspension of PFs (PFs-suspension) before and after the blockade of the lymphatic transport route by cycloheximide, respectively. These results suggested PFs-SMEDDS_NR_ could significantly improve the oral relative absorption of PFs via the lymphatic uptake pathway. SMEDDS containing NaTC may provide an effective approach for enhancing the oral bioavailability of PFs.

## 1. Introduction

More than 50% of compounds currently in development belong to classes II and IV, which cover compounds with low solubility, in the Biopharmaceutical Classification System [1]. Dissolution is a rate-limiting step for the oral bioavailability of poorly water-soluble compounds [2,3], and numerous formulation approaches, such as salt formation, co-solvent systems, solid dispersion, prodrug formation, particle size reduction, and solid lipid nanoparticles, are used to improve their low solubility and dissolution rate [4,5,6].

Recently, self-emulsifying drug-delivery systems (SMEDDS) attracted much attention owing to their potential to facilitate the solubility and bioavailability of water-insoluble drugs in the pharmaceutical industry. SMEDDS are composed of oils, surfactants, and cosurfactants, which can spontaneously form transparent and stable microemulsion systems upon aqueous dilution under gentle agitation conditions [2,7,8]. The resultant nanosized droplets provide large interfacial surface areas that benefit drug release and absorption [4,9,10]. Therefore, SMEDDS may be a promising approach to enhancing the solubility and oral bioavailability of poorly water-soluble drugs. Some marketed examples from SMEDDS are clinically applied such as cyclosporine A (Sandimmune Neoral^®^), ritonavir (Norvir^®^), saquinavir (Fortovase^®^), tipranavir (Aptivus^®^), and lopinavir and ritonavir (Kaletra^®^) [2,4]. However, many surfactants used in SMEDDS may give rise to some gastrointestinal side effects [11,12]; several strategies were adopted to reduce the surfactant-related toxicity of SMEDDS [12,13]. In vivo pharmacokinetic results showed that supersaturated SMEDDS containing hydroxypropyl methylcellulose could promote oral drug bioavailability and reduce surfactant-related toxicity [13]. Replacing polysorbate 80, the surfactant for SMEDDS, with natural emulsifiers can reduce surfactant-related toxicity [12].

*Radix puerariae* is a traditional Chinese medicinal herb whose main components are pueraria flavones (PFs). PFs mainly include puerarin, mirificin, 3′-methoxyl-puerarin, daidzein, and daidzin, among which puerarin is the most active component. PFs reduce myocardial oxygen consumption, dilate coronary arteries, and enhance microcirculation [14,15]. Moreover, they are used for the treatment of diseases, such as senile ischemic cerebrovascular disease, hypercholesterolemia, angina pectoris, coronary heart disease, and angiocardiopathy [16,17]. However, their clinical effects are restricted because of their poor water solubility [18].

In our previous study [19], PF-loaded SMEDDS using sodium taurocholate (NaTC) and Cremophor RH 40 as mixed surfactants (PFs-SMEDDS_NR_) were prepared to decrease the surfactant-related cytotoxicity of SMEDDS. The cytotoxicity was evaluated using the 3-(4,5-dimethylthiazol-2-yl)-2,5-diphenyltetrazolium bromide (MTT) method and lactate dehydrogenase release assay. The results demonstrated that the toxicity of SMEDDS using NaTC and Cremophor RH 40 as mixed surfactants was lower than that of SMEDDS only using Cremophor RH 40 as a surfactant, at the same amount of surfactant [19]. Bile salts, such as sodium cholate (NaC), sodium deoxycholate (NaDC), and NaTC, are natural anionic steroidal biosurfactants that are deeply involved in various biological processes and are mainly found in the bile of mammals, including humans and other vertebrates [20,21]. Additionally, bile salts act as solubilizers and emulsifiers [20,21].

In the present study, SMEDDS containing NaTC, NaDC, or NaC were prepared and compared in terms of particle size, zeta potential, and loading content. Moreover, the in vitro release behavior of PFs-SMEDDS was studied. The pharmacokinetics and lymphatic transport of PFs-SMEDDS were also estimated after oral administration in rats.

## 2. Materials and Methods

### 2.1. Materials and Animals

Pueraria flavones (PFs) with a purity of 66.70% were bought from Xi’an Saibang Pharmaceuticals and Technology Co., Ltd. (Xi’an, China). Standard puerarin with a purity of 99.10% was supplied by the National Institute for the Control of Pharmaceutical and Biological Products (Beijing, China). Maisine 35-1 was supplied by Gattefosse (Lyon, France). Polyoxyl 40 hydrogenated castor oil (Cremophor RH40) was gifted by BASF (Ludwigshafen, Germany). Sodium taurocholate (NaTC), sodium deoxycholate (NaDC), and sodium cholate (NaC) were purchased from Shanghai Ryon Biological Technology Co., Ltd. (Shanghai, China). Porcine pancreas lipase was provided by Sigma (100–500 Units/mg, Sigma-Aldrich, St. Louis, MO, USA). Sodium carboxymethyl cellulose was purchased from Yingte Chemical Co., Ltd. (Shijiazhuang, China). The *p*-hydroxybenzoic acid was bought from Shanghai Hushi Laboratorial Equipment Co., Ltd. (Shanghai, China). Cycloheximide was purchased from Shanghai Yuanye Biological Technology Co., Ltd. (Shanghai, China). Meanwhile, 1,2-propylene glycol was supplied by Tianjin Guangfu Fine Chemical Research Institute (Tianjin, China). HPLC-grade methanol and acetonitrile were bought from Fisher Scientific (Waltham, MA, USA). Hydrochloric acid and sodium hydroxide were purchased from Beijing Chemical Works (Beijing, China); Potassium phosphate monobasic was purchased from Tianjin Guangfu Fine Chemical Research Institute (Tianjin, China). Deionized water was provided by Milli-Q (Millipore, Bedford, MA, USA). All other chemicals were of analytical grade in the study.

Male Wistar rats (250 ± 20 g) used in the experiment were provided by the Central Animal Laboratory of Jilin University (Changchun, China, certificate No. SCXK (Ji) 2013-002, 10 July 2013). All animals were fed at a room temperature of 25 ± 2 °C for six days before the experiment. All experiments were conducted according to the Guidelines for Care and Use of Laboratory Animals of Jilin University and the Principles of Laboratory Animal Care of Jilin University.

### 2.2. Preparation of Self-Emulsifying Drug-Delivery Systems (SMEDDS)

Blank SMEDDS were obtained by mixing oil, cosurfactant, and surfactant. Briefly, 10% Maisine 35-1 (oil phase), 30% Cremophor RH 40 and/or bile salts (surfactants), and 60% 1,2-propylene glycol (cosurfactant) were weighed and homogeneously mixed in a flask at 100 rpm at 37 ± 0.5 °C in a water bath. Cremophor RH 40 and/or bile salts (NaTC, NaDC, or NaC) were weighed at weight ratios of 10:0, 9:1, 8:2, 7:3, 6:4, 5:5, 4:6, 3:7, 2:8, 1:9, and 0:10. PFs-SMEDDS were obtained by adding PFs sieved through 80-mesh sieves into blank SMEDDS with gentle stirring in a 37 ± 0.5 °C water bath until a homogeneous mixture formed.

### 2.3. Preparation of PF Suspensions (Pueraria Flavones (PFs)-Suspension)

PFs were sieved through an 80-mesh sieve and uniformly dispersed in 0.3% (*w*/*v*) carboxymethyl cellulose (CMC)-Na solution to form a suspension with a concentration of 200 µg/mL. General shaking was necessary to make the suspension uniform before use.

### 2.4. Particle Size and Zeta Potential

Particle size and zeta potential were measured on a dynamic light scattering particle size analyzer (Zetasizer Nano ZS, Malvern Panalytical, Malvern, UK) with a wavelength of 633 nm at 25 °C after a 100-fold dilution (W_SMEDDS_: V_dilution media_ = 1:100, g:mL). In brief, 1 g of SMEDDS was placed in a flask and diluted with 100 mL of deionized water and/or other aqueous media. The flask was placed in a 37 ± 0.5 °C water bath and gently shaken to mix thoroughly. All experiments were carried out in triplicate. All data were expressed as the mean ± standard deviation (SD).

### 2.5. Determination of PFs

PFs are a mixture of several active ingredients involving puerarin, daidzin, daidzein, and puerarin-7-xyloside, among which puerarin is the most abundant and major active ingredient. Moreover, puerarin is always analyzed as the representative component of PFs [15,22]. In the present study, high-performance liquid chromatography (HPLC) was employed to measure the loading content of PFs in SMEDDS, PF release in vitro, bioavailability, and a lymphatic transport study of PFs in rat plasma. The LC-20AT (Shimadzu, Kyoto, Japan) system consisted of an LC-20AT pump and SPD-20A UV detector controlled by Lab-solution software. A Diamonsil C_18_ column (4.6 mm × 250 mm, 5 µm, Dikma, Beijing, China) was used. The mobile phase of isometric elution was composed of acetonitrile and distilled water with 0.05% acetic acid at a volume ratio of 12:88. The flow rate was constant at 1 mL/min, and the wavelength was set at 250 nm.

### 2.6. Loading Content (LC) of PFs in SMEDDS

An excess amount of PFs was added into blank SMEDDS with or without bile salts, and was then shaken at 37 ± 0.5 °C for 72 h in a water bath until equilibrium was reached. Samples were centrifuged at 13,350× *g* for 5 min to remove the precipitate. The supernatant was harvested, diluted with methanol, and assayed via HPLC.

### 2.7. Release Study In Vitro

A dialysis bag [23] was used to determine in vitro release in 150 mL of simulated release medium (pH-1.2 hydrochloride solution and pH-6.8 phosphate buffer containing 20 mM NaTC and 500 U/mL porcine pancreas lipase) [24] using a dissolution apparatus (RC806, Tianda Tianfa Technology Co., Ltd., Tianjin, China). The temperature was maintained at 37 ± 0.5 °C, and the paddle-stirring speed was set to 100 rpm. Deionized water was added to cause PFs-SMEDDS to form a microemulsion at 100-rpm stirring speed before being placed inside dialysis bags [23]. Approximately 2 mL of microemulsion containing 5 mg of PFs was placed inside the dialysis bags (molecular weight cut-off (MWCO) = 8–12 kDa). The dialysis bags were then immersed in release medium, and 3 mL of the release medium was withdrawn at predesigned time points, before being promptly replaced by an equivalent volume of the release medium at 37 ± 0.5 °C. All samples were filtered through a 0.45-µm membrane filter, and PF concentration in the filtrate was determined by HPLC. The cumulative release of PFs was calculated using Equation (1). Sink conditions were maintained throughout the experimental period.

(1) $$Q=\frac{\left[V_{0}C_{i}+V_{t}\sum C_{\left(i-1\right)}\right]}{m}\times 100\%,$$

where *m* is the total amount of PFs in SMEDDS, *V*_0_ is the total volume of the release medium, *C_i_* is the concentration of PFs in filtrate determined using HPLC at time *i*, *Q* is the cumulative release percentage of PFs in samples at an individual time, and *V_t_* is the volume of sample at each time.

### 2.8. Oral Bioavailability and Lymphatic Transport

#### 2.8.1. Protocol Design

All rats were fasted for 24 h with free access to water, and were randomly divided into four groups (*n =* 5 in each group) to evaluate oral pharmacokinetics and lymphatic drug transport after receiving a single equal oral dose of PFs-SMEDDS_NR_ or PFs-suspension. Two groups (saline-pretreated rats) received 0.6 mg/mL saline intraperitoneally before gavage administration of PFs-SMEDDS_NR_ and PFs-suspension, which were used to evaluate the pharmacokinetics of PFs in non-lymphatic blocked rats. The other two groups (cycloheximide-pretreated rats) received 3 mg/kg cycloheximide solution in normal saline (0.6 mg/mL) intraperitoneally before gavage administration of PFs-SMEDDS_NR_ and PFs-suspension, which were used to evaluate the pharmacokinetics of PFs-SMEDDS_NR_ and PFs suspension after lymphatic absorption pathways were blocked [25,26]. After 1 h, all rats received a single oral dose of PFs-suspension or PFs-SMEDDS_NR_ (PFs 116 mg/kg) via oral gavage. Blood (0.2 mL) was collected from the eyes using heparinized tubes at 5, 15, 30, 60, 120, 180, 240, and 360 min after oral administration. All samples were stored at −20 °C until analysis. All animals were sacrificed after the experiments.

#### 2.8.2. Processing of Blood Samples

Cold methanol (150 µL) and 20 µg/mL *p*-hydroxybenzoic acid solution (internal standard, 25 µL) were added to blood plasma (50 µL), vigorously vortexed for 2 min, and centrifuged at 5006× *g* for 10 min. The organic layer was collected and blow-dried using nitrogen. The resultant residue was dissolved with 200 µL of mobile phase, followed by centrifugation at 13,350× *g* for 10 min. An aliquot (20 µL) of the supernatant was injected into the HPLC system for analysis.

#### 2.8.3. Calculation of Pharmacokinetic Parameters and Data Analysis

Pharmacokinetic parameters, including the maximum plasma concentration (*C*_max_), area under the plasma concentration-time curve (AUC), time to reach maximum plasma concentration (*T*_max_), and mean retention time (MRT), were calculated using the DAS 2.1 pharmacokinetic software (issued by the State Food and Drug Administration of China). The PF fraction transported to the lymphatic system was evaluated based on the difference between its bioavailability in rats pretreated with cycloheximide and that in rats pretreated with saline. Experiments were performed in triplicate, and data were expressed as mean ± SD. Statistical significance was tested using a Student’s *t*-test or one-way ANOVA using SPSS 19.0 (International Business Machines Co., Ltd., New York, NY, USA).

## 3. Results

### 3.1. Self-Microemulsifying Behavior

The high surfactant hydrophilic lipophilic balance (HLB) value is a key factor for the immediate formation of emulsion with small droplets in the aqueous environment, providing a good self-emulsifying performance [27]. Generally, the surfactants used in SMEDDS have high HLB values (9–20) [28]. The HLB value of Cremeophor RH40 is 12–14 [29], and the average HLB value of Cremeophor RH 40 is 13. The HLB values of NaTC, NaDC, and NaC are 39.175, 21.925, and 21.825, respectively, based on the calculation of numbers of hydrophilic groups and lipophilic groups [30]. The HLB values of mixed surfactants (*HLB_mix_*) were calculated using weight fractions of the orresponding surfactants, as shown in Equation (2) [31].

(2) $$HLB_{mix}=f_{A}HLB_{A}+f_{B}HLB_{B},$$

where *HLB_A_* and *HLB_B_* are the HLB values of Cremeophor 40 and bile salts, and *f_A_* and *f_B_* are the weight fractions of Cremeophor 40 and bile salts, respectively.

The mixed HLB values of Cremeophor 40/bile salts were in the range of 13–39.18. SMEDDS using Cremophor RH 40 as a surfactant without bile salts possessed good self-microemulsifying performance after a 100-fold dilution with deionized water, and retained a transparent blue–opalescent appearance. Moreover, SMEDDS exhibited good clarity and self-microemulsifying property at Cremophor RH 40:NaTC, Cremophor RH 40:NaDC, or Cremophor RH 40:NaC mass ratios of 9:1 to 2:8, 9:1 to 2:8, or 9:1 to 3:7, respectively, whereas white flocculent precipitates were observed at Cremophor RH 40:NaDC or Cremophor RH 40:NaC mass ratios of 1:9 or 2:8, respectively. These results implied SMEDDS could not spontaneously form transparent and stable microemulsion systems upon aqueous dilution any longer beyond Cremophor RH 40:NaTC, Cremophor RH 40:NaDC, or Cremophor RH 40:NaC mass ratios of 2:8, 2:8, and 3:7, respectvely.

### 3.2. Droplet Size and Zeta Potential of Blank SMEDDS

**Figure 1:**
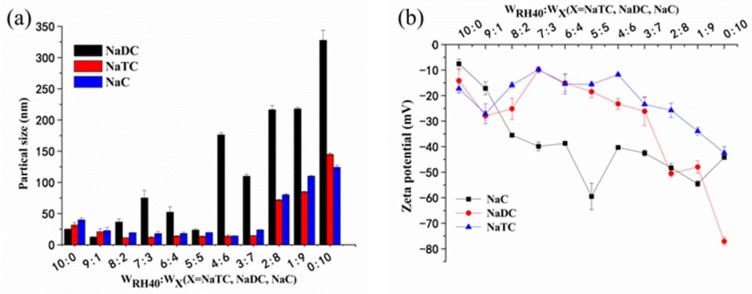
(**a**) Particle sizes and (**b**) zeta potentials of blank self-microemulsifying drug-delivery systems (SMEDDS) after being replaced with Cremophor RH 40 using bile salts (sodium deoxycholate (NaDC), sodium taurocholate (NaTC), or sodium cholate (NaC)) at different mass proportions of the mixed emulsifiers (mean ± SD, *n* = 3).

Droplet size is an important parameter in the development of SMEDDS because it influences the drug release rate, stability, and absorption. Figure 1a shows the effects of bile salts on particle sizes of blank SMEDDS under Cremophor RH 40: bile salt mass ratios of 10:0–0:10 (*w*/*w*) after a 100-fold dilution with deionized water. No distinct changes in the particle sizes of blank SMEDDS were observed at mass ratios of Cremophor RH 40:NaTC or Cremophor RH 40:NaC ranging from 9:1 to 3:7; however, the sizes abruptly increased at the weight percent of 80% NaTC or NaC (*w*_RH40_:*w*_NaTC or NaC_ = 2:8). The particle size of SMEDDS with NaDC was larger than that of SMEDDS with NaTC or NaC. The effects of NaTC, NaDC, or NaC on the zeta potentials of SMEDDS are shown in Figure 1b. Results proved that SMEDDS containing bile salts still carried a negative charge. However, the zeta potentials displayed irregular changes, as shown in Figure 1b. SMEDDS containing NaC carried a highly negative charge as the NaC mass ratio increased, which was mainly due to the high negative charge of NaC [21].

### 3.3. LC in SMEDDS

**Figure 2:**
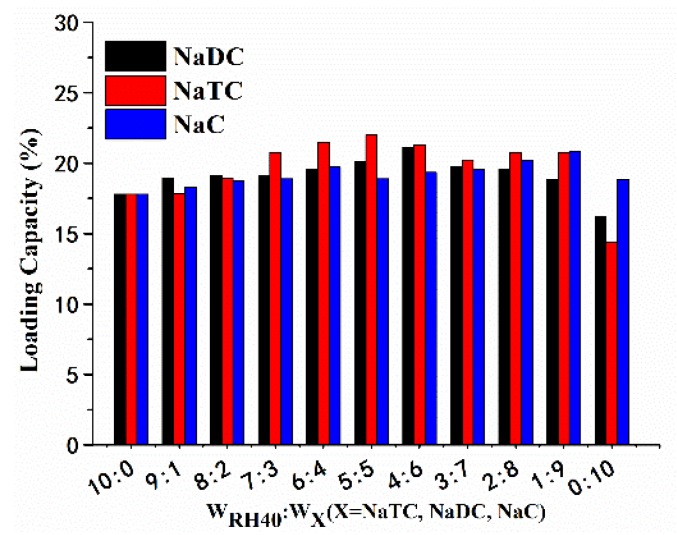
Loading capacities of blank SMEDDS after replacing RH40 using bile salts at different proportions of the mixed surfactants (*n* = 3).

The LC was measured using the HPLC system and the results are shown in Figure 2. LC increased with rising weight of NaDC or NaTC in mixed surfactants, and peaked at 20.09% and 21.26% at the mass ratios of 5:5 (Cremophor RH 40:NaTC) and 4:6 (Cremophor RH 40:NaDC), respectively. However, LC changed irregularly after Cremophor RH 40 was partly replaced by NaC in PFs-SMEDDS.

### 3.4. Mean Particle Size and Zeta Potential of PFs-SMEDDS

**Figure 3:**
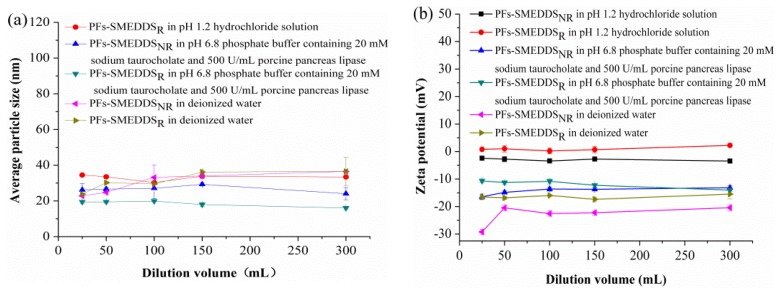
Effects of the volume of different media on average particles sizes (**a**) and zeta potentials (**b**) of pueraria flavone (PF)-loaded SMEDDS using NaTC and Cremophor RH 40 as mixed surfactants (PFs-SMEDDS_NR_) and with Cremophor RH 40 only (PFs-SMEDDS_R_) (mean ± SD, *n* = 3).

NaTC was selected to partly replace Cremophor RH 40 as a surfactant at a mass ratio of 5:5 based on the self-microemulsifying behavior, particle size, and LC of SMEDDS. PFs-SMEDDS_NR_ were prepared with the following mass ratio: 10% PFs, 9.0% Maisine 35-1, 13.5% Cremophor RH 40, 13.5% NaTC, and 54% 1,2-propylene glycol. The different dilution volumes of deionized water, pH-1.2 hydrochloride solution, and pH-6.8 phosphate buffer containing 20 mM NaTC and 500 U/mL porcine pancreas lipase along with the particle sizes of PFs-SMEDDS_NR_ and PFs-SMEDDS_R_ (Cremophor RH 40 with no bile salts) were used to stimulate dilution behavior in vivo. The particle size of PFs-SMEDDS_NR_ in pH-1.2 hydrochloride solution is not presented in Figure 3a because its particle size exceeded 1 μm. Figure 3a,b reveal that the dilution volume within the experimental range only slightly influenced particle sizes and zeta potentials. Figure 3a shows that PFs-SMEDDS_R_ appeared to have small particles in pH-6.8 phosphate buffer containing 20 mM NaTC and 500 U/mL porcine pancreas lipase. The particle size of SMEDDS_R_ was smaller than that of SMEDDS_NR_. The different dilution media greatly influenced particle sizes and zeta potentials (Figure 3b). The zeta potential of PFs-SMEDDS_NR_ carried highly negative charges in pH-6.8 phosphate buffer containing 20 mM NaTC and 500 U/mL porcine pancreas lipase and deionized water, whereas the zeta potential values of PFs-SMEDDS_NR_ and PFs-SMEDDS_R_ were close to neutralization, which was mainly attributed to the physicochemical characteristics of NaTC. NaTC is a weak acid and its apparent pKa value is different in diverse molecular environments [21].

**Figure 4:**
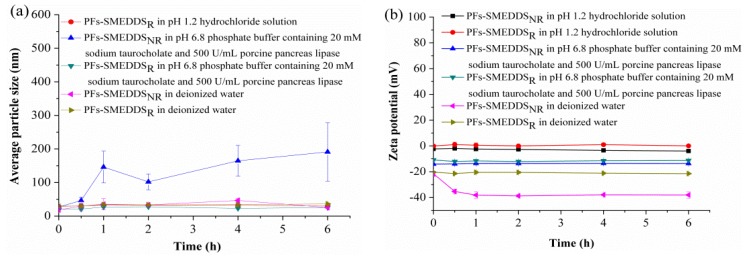
Effects of different media on average particle sizes (**a**) and zeta potentials (**b**) of PFs-SMEDDS_NR_ and PFs-SMEDDS_R_ (mean ± SD, *n* = 3).

The stabilities in terms of particle size of PFs-SMEDDS_NR_ or PFs-SMEDDS_R_ were determined after being diluted with pH-1.2 hydrochloride solution, pH-6.8 phosphate buffer solution containing 20 mM NaTC and 500 U/mL porcine pancreas lipase, and deionized water after a 100-fold dilution (*w*_PFs-SMEDDS_:*v*_dilution media_ = 1:100, g:mL), respectively. The particle size of PFs-SMEDDS_NR_ in pH-1.2 hydrochloride acid solution was not included in Figure 4a because its particle size exceeded 1 μm. Thus, PFs-SMEDDS_R_ presented similar particle sizes, but higher stability than PFs-SMEDDS_NR_, as shown in Figure 4a. Different dilution volumes had no obvious effects on zeta potentials within the experimental storage time, as shown in Figure 4b.

### 3.5. In Vitro Release Rate

**Figure 5:**
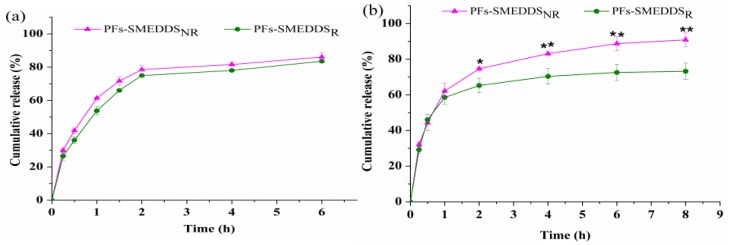
Release profiles of PFs from PFs-SMEDDS_NR_ and PFs-SMEDDS_R_ formulations determined in two different media: (**a**) pH-1.2 hydrochloride solution; (**b**) pH-6.8 phosphate buffer containing 20 mM NaTC and 500 U/mL porcine pancreas lipase (mean ± SD, *n* = 3). Asterisks indicate significant differences between PFs-SMEDDS_NR_ and PFs-SMEDDS_R_; * *p* < 0.05 and ** *p* < 0.01.

Diluting SMEDDS in water is crucial before placing them into dialysis bags to ensure precise and accurate measurement of the release rate [23]. SMEDDS can stick to the dialysis bag and restrict the inflow of release media. Therefore, deionized water was added to make PFs-SMEDDS self-microemulsifying before being placed inside dialysis bags at 37 °C and 100-rpm stirring speed. The in vitro release profiles of PFs-SMEDDS_R_ and PFs-SMEDDS_NR_ in pH-1.2 hydrochloride solution and pH-6.8 phosphate buffer solution containing 20 mM NaTC and 500 U/mL porcine pancreas lipase are shown in Figure 5. The release percentage of PFs increased with the extended release time. No remarkable differences between the PF release rates from PFs-SMEDDS_NR_ and that from PFs-SMEDDS_R_ in pH-1.2 hydrochloride solution were observed (Figure 5a), which were different from the release behavior in pH-6.8 phosphate buffer solution containing 20 mM NaTC and 500 U/mL porcine pancreas lipase (Figure 5b)_._ The PF release rate from PFs-SMEDDS_NR_ was significantly faster than that from PFs-SMEDDS_R_ (*p* < 0.05 or *p* < 0.01). The release percentages of PFs from SMEDDS_R_ and SMEDDS_NR_ at 8 h were 73.19 ± 4.57% and 90.84 ± 3.86%, which were likely due to the partial hydrolyzation of the lipids to free fatty acids by porcine pancreas lipase; this process further enhanced emulsification and micelle formation with NaTC [21].

### 3.6. Oral Bioavailability and Lymphatic Transport

#### 3.6.1. Determination of PFs in Plasma

The in vivo pharmacokinetic and lymphatic transport studies of PFs-SMEDDS_NR_ were performed using PFs-suspension as a control. The success of the PF assay in pharmacokinetic studies depends on the determination of the puerarin content [15]. In this study, the concentration of puerarin in rat plasma was measured using HPLC, as described in Section 2.5. No endogenous species interfered with puerarin analysis. The validities of the HPLC assay for puerarin and hydroxybenzoic acid (internal standard) were established. The retention time of puerarin was 13.84 min, whereas that of hydroxybenzoic acid (internal standard) was 15.67 min in the plasma sample. The peak area ratio of puerarin to hydroxybenzoic acid (*A*/*A*_i_) and puerarin concentration (*C*) exhibited a linear relationship at concentrations ranging from 0.1 µg/mL to 5.00 µg/mL, in accordance with the following equation: *A*/*A*_i_ = 0.0778*C* − 0.0409 (*R*^2^ = 0.9993). The mean relative SD of intraday and interday was ˂5%. The extraction efficiency of puerarin was 90.67–94.40%. The limits of detection and quantification were 30 and 10 ng/mL, respectively. All assays were performed in triplicate.

#### 3.6.2. Pharmacokinetics

**Figure 6:**
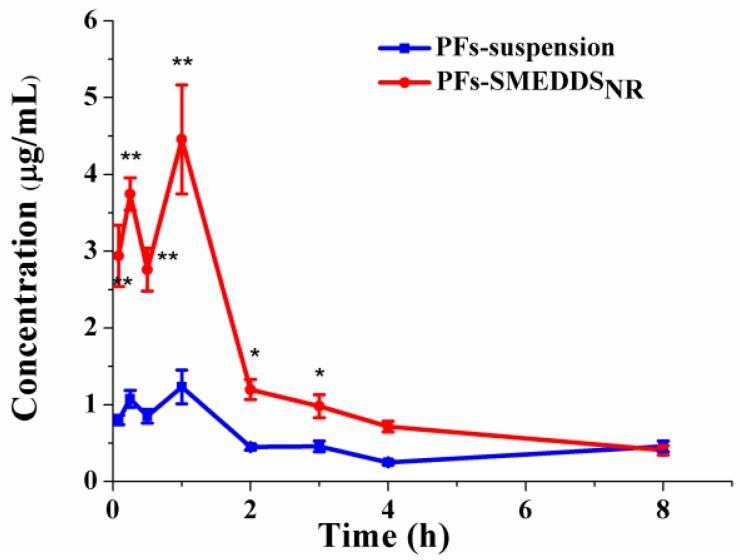
Plasma concentration–time profiles for oral administration of a suspension of PFs (PFs-suspension) and PFs-SMEDDS_NR_ (116 mg/mL) in saline-pretreated rats (mean ± SD, *n* = 5; * *p* < 0.05 and ** *p* < 0.01).

The plasma concentration-time profiles of PFs-suspension and PFs-SMEDDS_NR_ after oral administration in saline-pretreated rats are shown in Figure 6. The plasma concentration of PFs-SMEDDS_NR_ was significantly higher than that of the PFs-suspension (*p* < 0.01 or *p* < 0.05). The area under the plasma concentration-time curve (AUC_0→__8_) of PFs-SMEDDS_NR_ was remarkably higher than that of the PFs-suspension. The relatively enhanced bioavailability of PFs-SMEDDS_NR_ may be attributed to the enhanced drug absorption by improving the solubility and membrane permeability of the poorly soluble drugs in the gastrointestinal tract [32,33]. After oral administration, SMEDDS spontaneously form tiny emulsion droplets with a diameter of ˂100 nm under physiological temperature and gastrointestinal peristalsis. Microemulsions possess a large surface area, which can increase the permeability of drugs into gastrointestinal epithelial cells [2]. Microemulsions may facilitate drug oral bioavailability via the lymphatic transport pathway [34]. Moreover, bile salts can enhance the intestinal absorption of orally administrated drugs by interacting with the drug, resulting in drug incorporation or alteration in the permeability of the biological membrane [35]. Drug absorption showed obvious “double peaks”, which may be due to two reasons: (1) gastric and intestinal absorption, and (2) the enterohepatic cycle. The gastrointestinal tract is a complex system; the first organ which a drug encounters after oral administration is the stomach, which involves many digestive enzymes, and displays an acidic environment (pH 1.5–3.5); very few drugs are absorbed through stomach [36]. A phenolic hydroxyl group in the molecular structure of puerarin displays weak acidity. In the stomach environment, PFs are in a non-dissociated state, which can facilitate PF absorption to a certain extent. In our previous report, the results of an in situ stomach perfusion experiment showed that 10.6% of PFs were absorbed in the stomach [19]. The small intestine (duodenum, jejunum, and ileum) is the major site for drug absorption [36]. However, the majority of PFs were absorbed in the small intestine because of the physical performance of the small intestine. Gastric and intestinal absorption resulted in the formation of a double absorption peak. After a drug is ingested, it is secreted into the gallbladder in the form of a prototype or a metabolite, before accumulating in the gallbladder, entering the intestine through the common bile duct, and partially being absorbed by the bacterial group in the intestinal tract to form an enterohepatic cycle.

The pharmacokinetic parameters are listed in Table 1. The values of *C*_max_ (4.46 ± 0.71 μg/mL) and AUC_0→__8_ (618.67 ± 47.99 μg·min/mL) of PFs-SMEDDS_NR_ were considerably higher than those of PFs-suspension (*C*_max_ = 1.23 ± 0.22 μg/mL, and AUC_0→8_ = 240.73 ± 16.54 μg·min/mL; *p* < 0.01). The AUC_0→__8_ of PFs-SMEDDS_NR_ was remarkably higher than that of the PFs-suspension (*p* < 0.01) with a relative bioavailability of 257.00%. The results demonstrated that PFs-SMEDDS_NR_ could significantly enhance the oral absorption of PFs. These results were consistent with the findings of several previous studies [19,37,38].

#### 3.6.3. Effects of Lymphatic Transport on the Oral Absorption of PFs

Lymphatic transport can be directly estimated by surgically inserting a catheter into the rat lymphatic vessel to collect lymph fluid. However, this method requires a high level of surgical skill, and its success rate is relatively very low. Recently, a method was developed to study lymphatic transport without the cannulation of lymph ducts, using cycloheximide to block chylomicron flow [39]. The effects of the cycloheximide model and mesenteric lymph duct cannulated rat model on the absorption of vitamin D_3_ were evaluated without affecting the non-lymphatic absorption routes, and without leading to systemic side effects [39]. The cycloheximide model can be used to investigate lymphatic transport. Hence, in this study, we evaluated lymphatic transport via the chemical blockade of chylomicron flow with cycloheximide.

**Figure 7:**
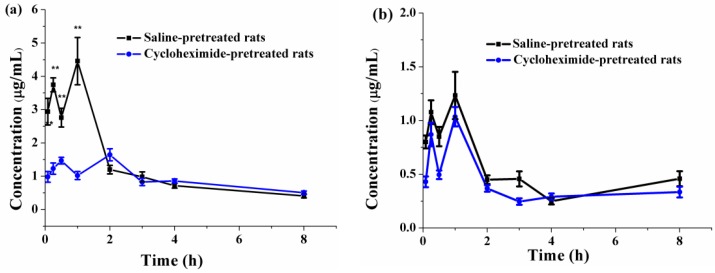
Plasma concentration–time profiles for oral administration of (**a**) PFs-SMEDDS_NR_ and (**b**) PFs-suspension in cycloheximide-pretreated and saline-pretreated rats (mean ± SD, *n* = 5; ** *p* < 0.01).

Oral lymphatic transport absorption can be evaluated by comparing the pharmacokinetic parameters of PFs in saline-pretreated rats and cycloheximide-pretreated rats. The pharmacokinetic parameters of PFs-SMEDDS_NR_ and PFs-suspension in rats receiving 3 mg/kg cycloheximide or an equal volume of saline are listed in Table 2. The *C*_max_ (1.67 ± 0.15 μg/mL) and AUC_0__→__8_ (438.50 ± 16.90 μg·min/mL) of PFs-SMEDDS_NR_ were considerably higher than those of PFs-suspension (*C*_max_ = 1.03 ± 0.09 μg/mL and AUC_0__→__8_ = 192.29 ± 10.06 μg·min/mL; *p* < 0.01). Comparing the pharmacokinetic parameters of PFs-SMEDDS_NR_ and PFs-suspension in Table 1 and Table 2, no significant differences were observed in *T*_max_ (1.00 ± 0.00 vs. 1.00 ± 0.00 h), *C*_max_ (1.23 ± 0.22 vs. 1.03 ± 0.09 μg/mL), and MRT _0→8_ (198 ± 9.65 vs. 199.02 ± 9.48) of PFs-suspension between the saline-pretreated groups and cycloheximide-pretreated groups (*p* > 0.05). However, AUC_0→8_ (192.29 ± 10.06 μg·min/mL) of PFs-suspension in the cycloheximide-pretreated group was much lower than that in the saline-pretreated group (240.73 ± 16.54 μg·min/mL; *p* < 0.01), which suggested that PFs might be partly absorbed via the lymphatic transport route. The *T*_max_ and MRT_0→8_ of PFs-SMEDDS_NR_ in the cycloheximide-pretreated group (1.50 ± 0.77 h and 193.08 ± 3.27 min, respectively) were significantly extended in comparison with those in the saline-pretreated rats (1.00 ± 0.00 h and 134.55 ± 8.51 min, respectively; *p* < 0.05 or *p* < 0.01). The *C*_max_ of PFs-SMEDDS_NR_ in the cycloheximide-pretreated rats dropped to 1.67 ± 0.15 μg/mL, which was significantly lower than that in the saline-pretreated group (4.46 ± 0.71 μg/mL; *p* < 0.05). The AUC_0*→*t_ *of* PFs-SMEDDS_NR_ remarkably decreased from 618.67 ± 47.99 μg·min/mL to 438.5 ± 16.9 μg·min/mL after blocking the lymphatic transport pathway (*p* < 0.01). The relative bioavailability of PFs-SMEDDS_NR_ in the saline-pretreated rats and cycloheximide-pretreated rats was 257.00% and 228.04%, respectively, indicating that it decreased by 29.12% after lymphatic transport was blocked by cycloheximide. The plasma concentration–time profiles of PFs-SMEDDS_NR_ and PFs-suspension in cycloheximide-pretreated rats are exhibited in Figure 7. PFs-SMEDDS_NR_ and PFs-suspension in cycloheximide-pretreated rats still exhibited obvious “double peaks”. The AUC_0→8_ value of PFs-SMEDDS_NR_ obviously decreased after blocking the lymphatic transport pathway, as shown in Figure 7a; the results demonstrated the lymphatic transport of SMEDDS_NR_ also facilitated the absorption of PFs and enhanced its oral bioavailability. However, the AUC_0→8_ value of PFs-suspension was slightly reduced after blocking the lymphatic transport pathway, as shown in Figure 7b.

## 4. Conclusions

SMEDDS containing bile salts (e.g., NaTC, NaDC, or NaC) were prepared, and their physical properties (i.e., self-microemulsifying characteristics, particle size, and LC) were compared. Blank SMEDDS with NaTC exhibited better self-microemulsifying characterics, smaller particle size, and higher loading drug capacity at a Cremophor RH 40: NaTC mass ratio of 5:5 than SMEDDSs with varying mass ratios of Cremophor RH 40 to NaTC, NaDC, or NaC. Our previous reports revealed that SMEDDS_NR_ can provide low surfactant-related cytotoxicity of SMEDDS, and NaTC can reduce the surfactant-related toxicity [20]. Therefore, PFs-SMEDDS_NR_ were prepared for further investigation in the study. Dilution volume had minimal effects on particle sizes and zeta potentials of PFs-SMEDDS_NR_ and PFs-SMEDDS_R_. The particle size of PFs-SMEDDS_R_ was relatively smaller and more stable in the same dilution media than PFs-SMEDDS_NR_. However, PFs-SMEDDS_NR_ showed a faster release rate in vitro than PFs-SMEDDS_R_. The oral bioavailability of PFs-SMEDDS_NR_ was dramatically enhanced by approximately 2.57- and 2.28-fold in saline-pretreated rats and cycloheximide-pretreated rats, respectively, compared with that of the PFs-suspension. Oral bioavailability decreased after the lymphatic transport route was blocked in cycloheximide-pretreated rats. These results suggest that PFs-SMEDDS_NR_ could improve the relative oral absorption of PFs via the lymphatic uptake pathway. SMEDDS containing NaTC could enhance the oral bioavailability of PFs, and lymphatic transport was one of the reasons for facilitating the oral absorption of PFs in PFs-SMEDDS_NR_.

## Figures and Tables

**Table 1:** Pharmacokinetic parameters of oral administration in saline-pretreated rats at a dose of 116 mg/kg pueraria flavones (PFs) (*n* = 5). *C*_max_—maximum plasma concentration; *T*_max_—time to reach maximum plasma concentration; AUC—area under plasma concentration–time curve; MRT—mean retention time; PFs-SMEDDS_NR_—PF-loaded self-microemulsifying drug-delivery system (SMEDDS) using NaTC and Cremophor RH 40 as mixed surfactants; PFs-suspension—a suspension of PFs.

Parameters	PFs-Suspension	PFs-SMEDDS_NR_
*C*_max_ (μg/mL)	1.23 ± 0.22	4.46 ± 0.71 **
*T*_max_ (h)	1.00 ± 0.00	1.00 ± 0.00
AUC_0→__8_ (μg·min/mL)	240.73 ± 16.54	618.67 ± 47.99 **
MRT_0→__8_ (min)	198.00 ± 9.65	134.55 ± 8.51 **

Values are mean ± SD for *C*_max_, *T*_max_, AUC_0→8_, and MRT_0→8_. ** *p* < 0.01 vs. PFs-suspension.

**Table 2:** Pharmacokinetic parameters of oral administration in cycloheximide-pretreated rats at a dose of 116 mg/kg PFs (*n* = 5).

Parameters	PFs-Suspension	PFs-SMEDDS_NR_
*C*_max_ (μg/mL)	1.03 ± 0.09	1.67 ± 0.15 ^∆∆^
*T*_max_ (h)	1.00 ± 0.00	1.50 ± 0.77 ^∆^
AUC_0→__8_ (μg·min/mL)	192.29 ± 10.06	438.50 ± 16.90 ^∆∆^
MRT_0→__8_ (min)	199.02 ± 9.48	193.08 ± 3.27

Values are mean ± SD for *C*_max_, *T*_max_, AUC_0__→__8,_ and MRT_0__→__8_. ^∆^ *p* < 0.05 and ^∆∆^ *p* < 0.01 vs. PFs-suspension.
